# Explanation and Apology

**Published:** 1902-12

**Authors:** 


### EXPLANATION AND APOLOGY.

THE serious illness of the editor through several weeks has made the preparation of the present number somewhat difficult. Our readers and correspondents will, therefore, kindly overlook all defects and delays in noticing their communications. All matter requiring the editor's special care, such as illustrated papers and book reviews, are necessarily held until he is able to resume his duties.

